# ECG based apnea detection by multirate processing hybrid of wavelet-empirical decomposition Hjorth features extraction and neural networks

**DOI:** 10.1371/journal.pone.0293610

**Published:** 2023-11-02

**Authors:** Sarika Khandelwal, Nilima Salankar, Saeed Mian Qaisar, Jyoti Upadhyay, Paweł Pławiak

**Affiliations:** 1 CSE Department G H Raisoni College of Engineering, Nagpur, India; 2 Persistent Systems Limited, Nagpur, India; 3 Electrical and Computer Engineering Department, Effat University, Jeddah, Saudi Arabia; 4 CESI LINEACT, Lyon, France; 5 Department of Pharmaceutical Sciences, School of Health Sciences and Technology, University of Petroleum and Energy Studies Campus, Bidholi, Dehradun, India; 6 Department of Computer Science, Faculty of Computer Science and Telecommunications, Cracow University of Technology, Warszawska, Krakow, Poland; 7 Institute of Theoretical and Applied Informatics, Polish Academy of Sciences, Bałtycka, Gliwice, Poland; National University of Sciences and Technology, PAKISTAN

## Abstract

Sleep Apnea (SA) can cause health complications including heart stroke and neurological disorders. The Polysomnography (PSG) test can detect the severity of sleep disturbance. However, it is expensive and requires a dedicated sleep laboratory and expertise to examine the patients. Therefore, it is not available to a large population in developing countries. This leads to the development of cost-effective and automated patient examination methods for the detection of sleep apnea. This study suggests an approach of using the ECG signals to categorize sleep apnea. In this work, we have devised an original technique of feature space designing by intelligently hybridizing the multirate processing, a mix of wavelet-empirical mode decomposition (W-EMD), modes-based Hjorth features extraction, and Adam-based optimized Multilayer perceptron neural network (MLPNN) for automated categorization of apnea. A publicly available ECG dataset is used for evaluating the performance of the suggested approach. Experiments are performed for four different sub-bands of the considered ECG signals. For each selected sub-band, five "Intrinsic Mode Functions" (IMFs) are extracted. Onward, three Hjorth features: complexity, activity, and mobility are mined from each IMF. In this way, four feature sets are formed based on wavelet-driven selected sub-bands. The performance of optimized MLPNN, for the apnea categorization, is compared for each feature set. Five different evaluation parameters are used to assess the performance. For the same dataset, a systematic comparison with current state-of-the-artwork has been done. Results have shown a classification accuracy of 98.12%.

## 1. Introduction

### 1.1 Context of the study

Sleep Apnea (SA) is a respiratory disorder that leads to some serious health problems like heart stroke and neurological issues. Therefore timely diagnosis and appropriate treatment of SA is important. It is reported that around 936 million individuals are globally suffering from SA. They mainly belong to the age groups ranging from 30 years to 69 years including men and women. Of these, 426 million cases fall into the moderate or severe category [1].

The SA refers to a variety of breathing issues that occur during sleep and are linked to other health complications. It is distinguished by recurring episodes of decreased (hypopnea) or halted (apnea) airflow in the upper respiratory tract during sleep. This causes variations in the arterial oxygen supply leading to sleep fragmentation. The obstructive sleep apnea is mainly caused by impaired cardiovascular system, quality of life, metabolic syndrome and accidental injuries, still the etiology of this disorder remain undiagnosed [2].

To diagnose SA, the determination of the sleep stage cycle is very important. Sleep staging is performed to investigate sleep patterns and to determine total sleep time accurately. An accurate detection of total sleep time helps in determining the sleep apnea as it significantly affects all the parameters which assesses the severity of this problem [3]. The pathophysiology of SA varies considerably among individuals and is multifactorial. The probable risk factors associated with sleep apnea include aging, male sex, and obesity. Adipose tissue build-up around the pharyngeal airway is believed to increase the risk of pharyngeal airway collapse, while abdominal fat deposition lowers functional residual capacity. These changes cause reduced lung volume capacity affecting the upper airway tract. Reduced lung volume also depleted the oxygen stores that contribute to ventilatory control instability thus obesity is found to be an associated risk factor that causes functional impairment of upper respiratory airway tract muscles [4]. The increased prevalence of SA in men has also been linked to gender differences. In females, the propensity to develop SA increases after menopause. In some studies, it was observed that the administration of testosterone in hypogonadal men was found to induce sleep-related breathing disorders. Additionally, it was found that men have a stronger ventilatory reaction to waking from sleep than women [5]. Several studies reported apnea frequency increases with ageing and shows remarkable prevalence of SA in elderly. Genioglossus negative pressure reflex is found to get deteriorated with ageing and found to be associated with upper airway collapse [6]. In contrast to healthy people, SA sufferers are shown to have higher upper airway surface forces rather than similar salivary secretions [7–9]. It was also observed that SA patients shows protective reflexes of increased upper airway dilation by muscle activity which maintain airway patency. Genioglossus is the largest and most abundantly used dilator muscles in the upper airway tract in humans that shows higher activity in SA patients as compared to the control group. The important mechanism behind SA pathogenesis relates to the interaction between reduced ability of the dilator muscles of the upper airway tract and pharyngeal anatomy to regulate patent airway during sleep [10].

The Polysomnography (PSG) is used to measure the physiological signals to diagnose SA. It is performed under the supervision of clinicians. Although it is very important diagnostic instrument in sleep research but it is not easily affordable due to the high cost of equipment and required facilities. Therefore, it decreases its SA diagnostic potential particularly for developing countries [11].

### 1.2 The related works

The PSG test is the recognized standard for detecting sleep apnea [11]. It can detect the severity of the syndrome and also the extent of sleep disturbance. However, it is not available to a large group of the population as it requires a dedicated sleep laboratory and experienced medical specialists. Few other simple and cost-efficient methods to detect sleep apnea involve cardiorespiratory monitoring or oximeter monitoring. These procedures, however, need the patient to wear those diagnostic tools for at least one night. Also, the availability of these devices and the increase in the number of patients for SA may lead to long waiting queues [11]. Hence, there is a need for a simple and effective method to predict and detect SA.

In this context, researchers have proposed Artificial Intelligence (AI) assistive approaches for the automated detection of SA. In [11], Mencar et al., suggested a method for SA detection. The processed dataset of 313 SA patients has been categorized as per American Association of Sleep Medicine’s guidelines (AASM). Principal Component Analysis (PCA) is used for feature selection. The selected feature set was used to train and evaluate the classification and regression models while following the 10-fold cross-validation strategy. The classification model is used to classify the severity classes whereas the regression model is used for predicting the numerical Apnea Hypopnea Index (AHI) score values. The support Vector Machine (SVM) classifier was reported to have the highest classification accuracy value of 44.7%. The lowest root mean squared error of 22.17 was reported for the case of Support Vector Regression (SVR).

In [12], Kristiansen et al., studied the A3 dataset for the detection of SA using unattended sleep monitoring at home. 579 pre-processed recordings comprising 7408.85 hours of sleep data has been used for the subjects with Paroxysmal Atrial Fibrillation (PAF). Four different signals namely N, O, C, and A are monitored using NOX-T3 Device. This signal data is annotated with sleep scores by sleep experts as per guidelines in AASM. The Deep Neural Network (DNN) is applied to four signals as well as one signal. Using all four signal types concurrently, they were able to obtain an accuracy of 0.8941 (kappa: 0.7877), whereas using only one signal, oxygen saturation, they were able to reach an accuracy of up to 0.8543 (kappa: 0.7080). According to the observed outcomes, single-signal monitoring is a preferable choice for long-term monitoring. The best combination for patients who can employ SPO2 with even data loss among the other 27 classifiers under investigation appears to be CNN with O signal, which provides an accuracy of 0.8543 (kappa: > 0.7080).

In [13], Kandala et al., have used dataset from physionet single-lead ECG. DWT based features are used to detect obstructive sleep apnea classes using automated computer aided approach. This dataset contains 70 sleep ECG records collected with 100 Hz sampling rate. Three distinct features, namely PSD moments, waveform complexity measurements, and higher-order moments are extracted using these ECG sub bands. In order to get the optimum feature vector, correlation-based feature selection with particle swarm optimization (PSO) search strategy is applied. With application of PSO, 18 significant features are retained out of 32 available features. DWT based statistical features are fed to various classifiers for classification of the ECG data. These parameters have been used by the RF classifier to distinguish between segments of normal and apneic ECG. Wavelet-based features may effectively categorise Normal and ECG signals with superior classification metrics than the currently available state of the art approaches, as demonstrated by the proposed method’s attendance accuracy of 90%.

In [14], Sheta et al., has presented the approach to diagnose SA from ECG signals using deep learning and machine learning approach. The authors have used ECG dataset of Physionet’s CinC challenge-2000 database containing 70 primary records. ECG signal of every record is in between [25,200, 36,000] minutes. Nine different features like average heart rate, Mean R-R interval distance etc. are extracted from those signals. Authors have used seven different available classifiers which includes “k-nearest neighbours” (KNN), “Naïve Bayes” (NB), “Linear discriminate analysis” (LDA), SVM, and “Boosted Trees” (BT). Authors have also applied six classifier with hyper-parameter tuning in order to optimize internal parameters. Few of the models are “Decision Tree” (DT), “Naïve Bayes” (NB), and kNN. These 13 Machine Learning (ML) models along with 4 Deep Learning (DL) models are used to classify the apnea cases. Out of the study of deep learning methods it was concluded by the authors that a combination of Convolutional Neural Network (CNN) with Long Short Term Memory (LSTM) secures the highest accuracy of 0.9075, precision of 0.9148, F1-score of 0.9163, and Area under the ROC curve (AUC) of 0.9746. The analysis of the data shows that the CNN-LSTM model can provide a reliable SA diagnosis.

In [15], Xie et al., have suggested the method for real-time SA detection using the ECG and “Peripheral Oxygen Saturation” (Spo2) signals individually as well as in combination. Authors have used the “University College Dublin Sleep Apnea” (UCD) database. The dataset contains overnight PSG recordings of 25 sleep disordered breathing suspects. For classification and detection of SA both the signals are segmented in 1 minute duration. They have used 39 features of Spo2 and 11 features of ECG signals. These 50 features are conveyed to 10 different classifiers in combination as well as individually. Out of the experiments, when ECG signals are considered individually, resulted in a respective specificity, sensitivity, and accuracy of 90%, 40% and 70%. For the case, when features of Spo2 are uniquely considered the specificity, sensitivity, and accuracy of 90%, 60%, and 80% is respectively reported. By combining feature sets of Spo2 and ECG signals, the highest sensitivity of 87.03% is achieved with AdaBoost and Decision Stump classifier. The Bagging with REPTree has proved to achieve both the highest specificity (85.89%) and accuracy (84.40%).

Bahrami et al., in [16] have studied and implemented different ML and DL algorithms for detection of SA from the EGC signals. The PhysioNet Apnea-ECG Database v1.0.0 was used. It contains the data of 32 subjects. The dataset contains 70 recordings which are divided in four classes as A, B, C, and X as per AHI values. Seven features from time domain, 13 features of frequency domain and seven nonlinear features of heart rate variability (HRV) are extracted and used for the classification models performance testing. For dimensionality reduction the PCA is used. The fivefold stratified cross validation strategy is followed while evaluating the classification performance. It was concluded from the results of all the comparisons that the DL algorithms outperformed the ML ones. Among the DL models, the “Deep Recurrent Neural Network” (DRNN) performs better for short ECG segments than the CNN. It is reported that the Hybrid CNN-DRNN models can be used to get optimised results. One of the intended hybrid models (ZFNet-BiLSTM), secured the highest accuracy and specificity values of 92.27%, while ZFNet-GRU attained the best sensitivity (84.26%), and the VGG16-LSTM achieved the highest F-score (84.24%). Therefore, it is advised to use a hybrid deep neural network to identify SA using the ECG signals.

In [17], Wang et al., have developed time window (TW) Artificial Neural Network (ANN) which does not require prior knowledge how the training data is distributed. The ECG signals are pre-processed to get the RR intervals and R peak amplitudes using Hamiltonian algorithm. The model is constructed using 12 RR interval time domain features and 6 R peak amplitude frequency domain features. The devised TW ANN model secures the SA detection sensitivity, accuracy, specificity and AUC of 85.1%, 87.3%, 88.7% and 0.945 respectively. Also TW ANN has given better results compared to non TW ANN approach.

In [18], Tuncer et al., have suggested a decision support system to diagnose the SA. They have used the Pulse Transition Time (PTT) parameters extraction approach. Onward, the mined feature set is used for training and testing of the CNN classifier. The PSG Recordings of 100 individuals were collected to create dataset for this study. They achieved accuracy of 92.78%, precision values of 95.70% and specificity of 98.30%, respectively.

In [19], Urtnasan et al., have used single led ECG recordings for multiclass categorization of SA using the CNN classifier. They have studied the PSG data of 86 subjects, obtained from Embla N7000 amplifier system at Sleep Center of Samsung Medical Centre, Seoul, Korea. The sleep experts have labelled the data as per standards of the American Academy of Sleep Medicine (AASM) guidelines. The pre-processing of ECG recordings were done by using bandpass filter to remove noise. An optimal six-layer CNN model is trained on a training dataset of 45,096 instances and evaluated on a test dataset of 11,274 instances. The proposed model has achieved the F1-score of 93% and accuracy of 90.8%.The brief summary of the study carried out in the literature is given in the Table 5 along with the achieved accuracy.

### 1.3 Contribution

The primary contributions of this work are:

- To devise a novel hybridization of the multirate processing, mix of wavelet-empirical mode decomposition (W-EMD), Intrinsic Mode Functions (IMFs) based Hjorth features extraction, and Adam based optimized multilayer perceptron neural network (MLPNN) for an automated and accurate categorization of SA.
- Investigating at four different sub-bands level the impact of IMFs based Hjorth features extraction to diagnose the SA. It has given a clear understanding that the analysis of only the pertinent sub-bands is sufficient to categorize the SA.

The functioning steps are:

- Each intended ECG instance is splitted in sub-bands by using the Wavelet-Decomposition (WD).
- Four sub-bands are selected as delta, theta, alpha and beta. Delta sub band is of range 0–4 Hz, Theta is of range 4–8 Hz, Alpha is of range 8–16 Hz and Beta is of range 16–32 Hz.
- For each selected sub-band 5 IMFs are extracted.
- Onward, 3 Hjorth features namely, mobility, activity, and complexity are mined from each IMF.
- In this way 4 feature sets are formed on the basis of wavelet driven selected sub-bands.
- The performance of Adam based optimized MLPNN, for an automated apnea categorization, is compared for each feature set.
- Five evaluation measures (accuracy, F1-score, sensitivity, specificity, and Kappa index) are used to assess the performance of each feature set’s apnea categorization capability.

According to reviews of recent studies from the years 2012 to 2022, the apnea automated identification is carried out by using a variety of ways that are centred around a variety of biological signals. However, as per the authors best knowledge the ECG signal processing based apnea categorization is not carried out in the above mentioned numerated manner.

For the same dataset, a systematic comparison with current state-of-the-art work is also done. It is discovered that the suggested technique secures a comparable or better performance. It shows the reliability and robustness of the devised solution for classifying apnea by processing the ECG signals.

The rest of the paper is divided in following sections. A description of the chosen methodology is given in the materials and methods section. The findings of every experiment are presented along with a discussion in results and discussion section 3. Finally conclusion is made.

## 2. Material and methods

**Fig 1:**
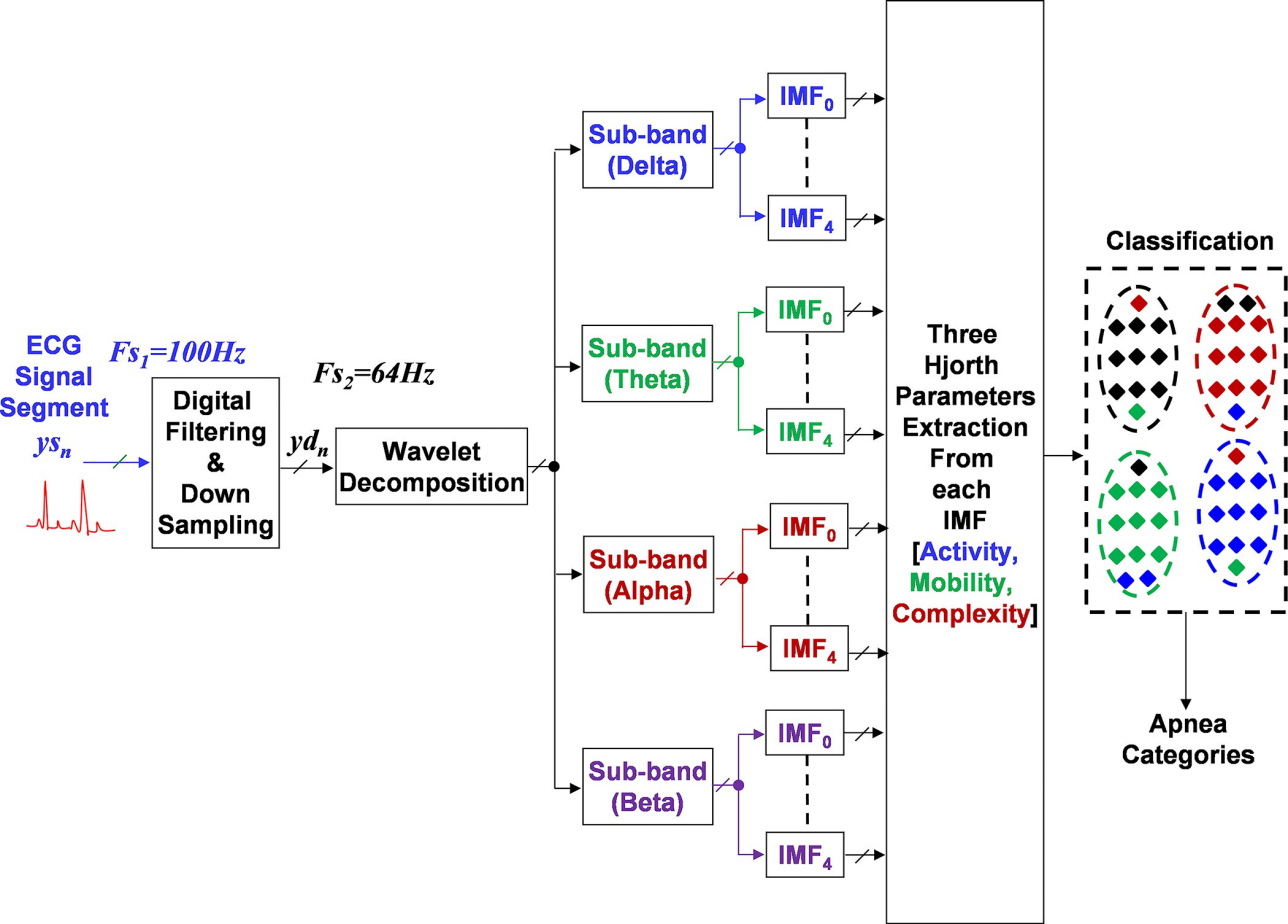
The block diagram of proposed method, showing the key processing stages used in this study.

The system block diagram of proposed method is shown in Fig 1. The sub-sections that follows provide descriptions of these phases.

Fig 1 shows that the devised scheme tactfully combines the wavelet decomposition with the Empirical Mode Decomposition (EMD). It offers a powerful approach for analysing and processing a variety of signals [20]. It is particularly beneficial in cases where the signals are complex, nonstationary, and contain multiple scales of information. The wavelet decomposition provides a multi-resolution representation of a signal, breaking it down in different frequency bands. The EMD is signal-driven, decomposing a signal in its oscillatory modes. This combination permits to capture both the frequency components highlighted by the wavelets and the oscillatory modes revealed by the EMD. Therefore, combining these features can yield a more comprehensive representation for the pattern recognition tasks.

### 2.1 Dataset

**Fig 2:**
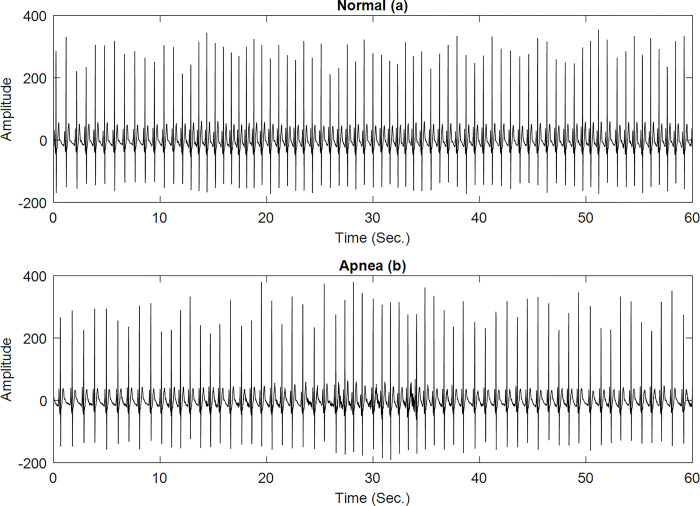
ECG signal examples in 60-second segments: (a) normal; (b) apnea class A.

The ECG recording can be helpful for diagnosing sleep apnea, According to Penzel et al. [21]. We used the ECG-apnea Dataset, which is freely available to the public, to ensure the accuracy of the proposed sleep apnea detection [22].The Dataset consist of ECG recordings of 70 records. Each ECG is recorded with sampling rate of 100Hz. Each recording includes a set of machine-generated QRS annotations, a set of apnea annotations, and continuous digitalized ECG Signals. Dataset is partitioned into learning set and test sets of 35 samples each. Each recording is varied from 401 to 578 minutes. The time spent in normal and disordered breathing was from 11-535mins and 0-534mins respectively. Based on the disordered breathing activity the database is categorized in three classes namely, A, B, and C. These classes are defined as per Apnea Hypopnea index score (AHI). Where class A is having AHI> = 10 which presents 10 or more SA segments per hour. Class B is having AHI > = 5 which indicates that number of SA segments per hour is 5 or more and class C is having AHI<5 which indicates that number of SA segments per hour is less than five [23]. The class C in this study is the normal class. This group consist of 11 people in the age group of 27–42. In this group the disorder breathing was in the range of 0-3mins. Group A or apnea is defined as the people whose recordings have more than 100mins of disordered breathing.16 such recording of the age group 27–63 was identified. Class B, a "borderline apnea" category with some apneas of unknown relevance, falls between these two groups. Ten recordings, each lasting 10 to 96 minutes, were included in this collection. Four males and one woman made up the subjects, who had a mean age of 46. (39–53 years). Each ECG sample duration that is considered is of 1 minute duration. Hence we have such 6511 Apnea ECG and 10454 Normal ECG samples to be used for training and testing. These ECG data are divided into fixed 60-second time segments. Each part of the signal comprises 6000 samples since the signal was initially captured at 100 Hz. The process of segmentation is carried out by using the rectangular window function, with the windowing operation given by: *ys*_*n*_ = *y*_*n*_×*s*_*n*_, here, *y*_*n*_ is the digitized ECG record, derived from the considered dataset [22]. *ys*_*n*_ is the segmented version of *y*_*n*_. *s*_*n*_ is the window function coefficients vector with a duration of *τ* = 60-Seconds and contains 6000 coefficients, each of magnitude 1. It leads towards a simplified presentation of the windowing process as: ${ys}_{n}=\sum_{\frac{-\tau }{2}}^{\frac{\tau }{2}}y_{n}$. Examples of normal and apnea ECG signals are shown in Fig 2.

### 2.2 Digital filtering and down sampling

The ECG signal should be denoised to remove the muscular artifacts and power line interference [24]. Therefore, a low-pass finite impulse response (FIR) filter is designed by using the Parks-McClellan algorithm. The result of denoising is an enhanced feature extraction and categorization. Additionally, it leads towards an effective breakdown of sub-bands [25]. The ECG signal’s useful spectrum is up to 30 Hz. Therefore, an offline implementation of a low-pass FIR filter with a cut-off frequency, *F*_*C*_ = 30 Hz and 100 Hz sampling frequency, *F*_*S*1_, is made. Coherent sampling frequencies between the input signal and filter are required for effective filtering [26]. Moreover, the selected sampling frequency should respect the condition: *F*_*S*1_≥2×*F*_*C*_ [27]. The filtering operation, for the studied case is given as: $x_{n}=\sum_{k=0}^{M-1}h_{k}\times {ys}_{n-k}$. Here, *x*_*n*_ is the filtered signal and *ys*_*n*_ is the segmented version of intended ECG record.

To get *yd*_*n*_ = *x*_*Dn*_, the *x*_*n*_ is down sampled with D = 1.5625. The cubic spline resampler is used to implement the fractional down sampling [28]. The selection of D = 1.5625 for this case complies with the requirement that $D\leq \frac{F_{S1}}{F_{Nyq}}=1.6667$ and does not, therefore, result in aliasing [27]. Here, *F*_*Nyq*_ = 2. *f*_*maxf*_, and *f*_*maxf*_, is *x*_*n*_’s maximum bandwidth, equals *F*_*C*_ = 30 Hz. By minimizing the quantity of data that has to be processed during the post decomposition and feature selection stages, this cascading step of subsampling promises a reduction the computational complexity of the system [27].

### 2.3 Wavelet decomposition

An effective feature extraction is realizable from the ECG like non-stationary signals’ multi-resolution analysis [27, 28]. Multi-resolution analysis typically employs the Wavelet Transform (WT).It might be formally expressed by Eq (1), where *u* and *s* notate the translation and dilation parameters, respectively:

$$W_{x}^{\psi }(u,s)=\frac{1}{\sqrt{S}}\int_{-\infty }^{+\infty }x(t)\psi *\left(\frac{\left(t-u\right)}{s}\right)dt.$$ (1)

**Fig 3:**
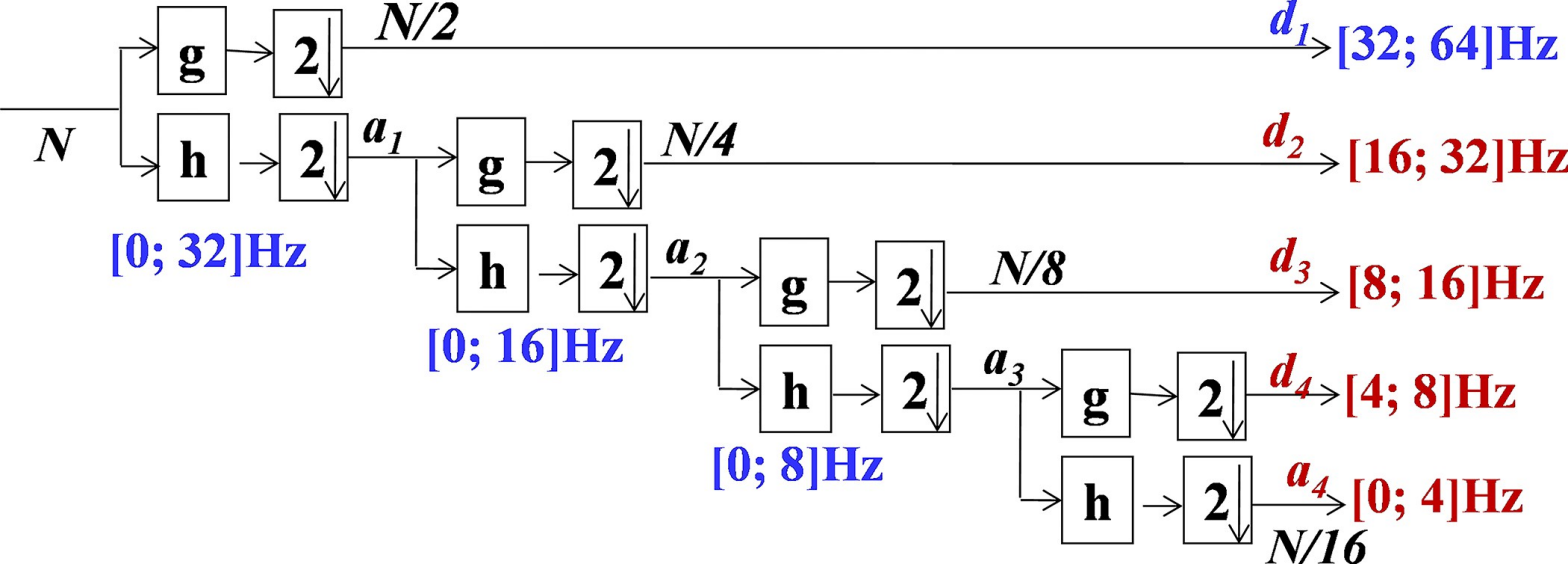
The employed wavelet decomposition scheme.

For the analysis of digital signals, the DWT is employed. The sub-bands decomposition is accomplished using half-band digital filters. The Daubechies algorithm-based wavelet is used for analysing the *yd*_*n*_. As a result, the sub-bands coefficients namely, the approximation (*a*_*m*_) and detail (*d*_*m*_) at each level (*m*), may be extracted by using Eq (2) and Eq (3). Where, the half-band low-pass and high-pass filters of length *M*+1 are respectively denoted by *g*_2*n*−*k*_ and *h*_2*n*−*k*_. The decomposition stages are shown in Fig 3.


$$a_{m}=\sum_{k=1}^{K_{g}}{xd}_{n}.g_{2n-k}.$$ (2)

$$d_{m}=\sum_{k=1}^{K_{g}}{xd}_{n}.h_{2n-k}.$$ (3)

**Fig 4:**
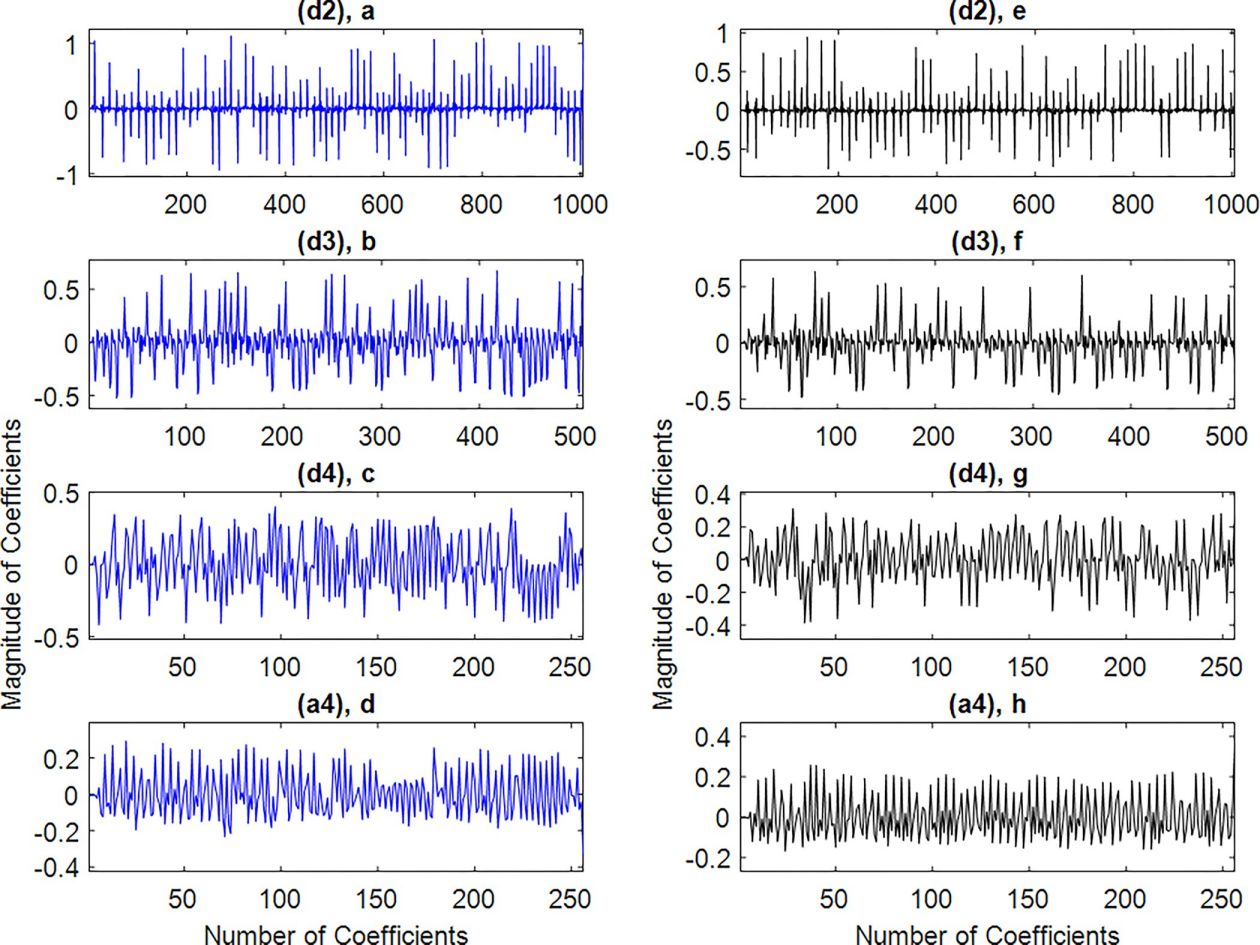
The selected sub-bands coefficients respectively for ECG normal (left-column) and ECG apnea (right-column).

The sub-bands *a4*, *d4*, *d3* and *d2* are selected in this study and are presented in red colour in Fig 3. Where, *a4*, *d4*, *d3*, and *d2* respectively present the Delta, Theta, Alpha, and Beta sub-bands. An example of these sub-bands is shown in Fig 4.

### 2.4 Empirical Mode Decomposition

The Empirical Mode Decomposition (EMD) is used to further analyze the chosen sub-bands: *a4*, *d4*, *d3* and *d2*. Eq (4) describes the EMD procedure of splitting a considered sub-band in five IMFs, *D*_*m*_*(t)*, and a residue, *r*_*4*_*(t)*.


$$z(t)=\sum_{m=0}^{4}D_{m}(t)+r_{4}(t).$$ (4)

In Eq (4), *z(t)* is an intended sub-band. In fact, the five IMFs and the residue can be added together to present *z(t)*.

IMFs are orthogonal to residue and to one another. Once the signal has a single peak and single trough then it can no longer decomposed. Otherwise, the process repeats iteratively unless the residue becomes monotonic [29]. In this study, the process of oscillatory modes decomposition is stopped after the 5^th^ iteration. The following six steps outline the full procedure.

- Find the minima and maxima of *z*(*t*).
- To determine the lower envelope *e*_*m*_(*t*), and the upper envelope, *e*_*l*_(*t*), perform the cubic spline interpolation of the identified minima and maxima respectively.
- To calculate average values of the two envelopes, perform the following calculation.
$$m(t)={(e}_{m}(t)+e_{l}(t))/2$$
- To find out *D*_*m*_(*t*), Compute the difference between *z(t)* and *m(t)*
$$D_{m}(t)=x(t)-m(t)$$
- Verify if *D*_*m*_(*t*) is an IMF or not [29, 30].
- If *D*_*m*_(*t*) is determined to be an IMF then find the residue, *r*(*t*) = *z*(*t*)−*D*_*m*_(*t*), and make sure the step-by-step requirements are satisfied. To find the next IMF, repeat steps (I) through (V) while treating the residue, *r(t)*, as input. If *D*_*m*_(*t*) is not an IMF then repeat steps (I) through (V) using *D*_*m*_(*t*) as input.

The *r*_4_(*t*) is considered as the ultimate residue in this study. After its computation, the EMD procedure is completed.

### 2.5 Features extraction

To diagnose a sleep apnea using computer aided method, feature extraction plays important role. To enhance the accuracy of the result, selection of appropriate features is important. By utilising the wavelet transform, the 30 Hz band limited ECG signal is divided in sub-bands. Four sub-bands are selected based on the frequency content. Five IMFs are then created from each band, and features are extracted; reason is to check for prominent band impact in classification.

In this study, three features are extracted from each IMF using Hjorth parameters. Those three extracted IMF features are activity, mobility and complexity [31]. Hjorth parameters quantify variance var of the ECG signal [32] and are respectively given by Eq (5)–Eq (7) [33].The activity parameter represents the signal power and the variance of a time function. Variance is a statistical measure which is used to give the amount of variability of a value with respect to its mean value among the set. Variance is calculated by calculating the average of squared deviation from the mean. This can indicate the surface of the power spectrum in the frequency domain. Activity parameter is given by Eq (5). The mobility, and complexity parameters represent the mean frequency and the standard deviation of the power spectrum, respectively. The mobility and complexity parameters are given in Eq (6) and Eq (7) respectively [34].

These three parameters are used as feature for ECG data. Statistical checks have been performed for each extracted features with p-value<0.01.


Activity=var(y(t)). (5)

$$Mobility=\sqrt{\frac{var(\frac{dy(t)}{dt})}{var(y(t))}}.$$ (6)

$$Complexity=\frac{Mobility(\frac{dy(t)}{dt})}{Mobility(y(t)}.$$ (7)

Where, y*(t)* is denoted as a signal and $var(\frac{dy(t)}{dt})$ *i*s the variance of first derivative of the signal

### 2.6 Classification

The classifier utilized is a multi-layer perceptron neural network that has been improved with Adam [35] for optimization. In order to improve the cost values and working on batch processing, Adam optimization based hyper tuning network is convenient. The main idea behind selecting Adam network is its fine-tuning flexibility and faster convergence. Optimizing the network with Adam is flexible as three parameter namely ε, β1 and β2 convergence of network is accurate and faster. Eqs 8–17 [36] describes the entire process involved in this optimization. Also, Adam is suitable for cost factor reduction during back propagation. It generalizes the training with smaller datasets, and thus over fitting issue can easily be handled hence network’s desirable behaviour can be achieved with low bias and low variance. In this work, the network is tuned with initial values of ε, β1 and β2 and as 1e^−8^, 0.9 and 0.999 respectively, learning rate α is set to 0.01 and initial values of S_dw_, S_db_, V_dw_, V_db_ = 0.Where β_1,_ β_2_ represents exponential decay rate for first moment estimates and second moment estimates respectively and ε is a small number to prevent any division by zero implementation.

The parameters of the model are W and b, where W is the weight and b is the bias term. The derivatives of the loss function with respect to W and b are dw and db. The exponentially weighted averages of the derivatives are V_dw_ and V_db_. V_dw_ is similar to momentum whereas S_dw_ is similar to RMSProp. S_dw_ and S_db_ are the exponentially weighted average of gradient squares. After correction of the bias we get $V_{dw}^{corrected},V_{db}^{corrected},S_{dw}^{corrected},S_{db}^{corrected}$ [12].


$$S_{dw}=\beta_{2}S_{dw}+(1‐\beta_{2})dw.$$ (8)

$$S_{db}=\beta_{2}S_{db}+(1‐\beta_{2})db.$$ (9)

$$V_{dw}=\beta_{1}V_{dw}+(1‐\beta_{1})dw.$$ (10)

$$V_{db}=\beta_{1}V_{db}+(1‐\beta_{1})db.$$ (11)

$$S_{dw}^{corrected}=\frac{Sdw}{1-\beta 2}.$$ (12)

$$S_{db}^{corrected}=\frac{Sdb}{1-\beta 2}.$$ (13)

$$V_{dw}^{corrected}=\frac{Sdw}{1-\beta 1}.$$ (14)

$$V_{db}^{corrected}=\frac{Sdb}{1-\beta 1}.$$ (15)

$$W=W-\alpha \frac{V_{dw}^{corrected}}{\sqrt{S_{dw}^{corrected}+\varepsilon }}.$$ (16)

$$b=b-\alpha \frac{V_{db}^{corrected}}{\sqrt{S_{db}^{corrected}+\varepsilon }}.$$ (17)

### 2.7 Evaluation measures

Sensitivity, Accuracy, specificity, F1-score, and kappa statistics are used to quantify the effectiveness of the suggested apnea classification method as described in [37]. Here, accuracy is the total number of both SA and normal ECG segments that were properly recognized and is represented by Eq (18).The number of successfully recognized SA from all SA ECG segments is reported by sensitivity and is given by Eq (19), and specificity report the correctly identified normal from total normal ECG segments and is represented by Eq (20) [13]. Kappa statistics (KI) and F1-score balances between precision and sensitivity. Where Precision is the measure of positive prediction and is given by Eq (21). Kappa index is most widely used statistics to judge agreement of two clustering results. As Kappa takes into consideration about agreement by chance into account hence it is considered to be better than accuracy. Kappa index is given by Eq (22). Higher kappa value indicates that the classification is good whereas zero kappa is indicator of classification by chance [38].


$$Accuracy(Acc)=\frac{\left(TP+TN\right)}{\left(TP+TN+FP+FN\right)}\times 100.$$ (18)

Where TN represents True Negative, TP represents True Positive, FN is used for False Negative and FP represents False Positive

$$Sensitivity(Sen)=\frac{TP}{\left(TP+FN\right)}*100.$$ (19)

$$Specificity(Spec)=\frac{TN}{\left(TN+FP\right)}*100.$$ (20)

$$F1-score=\frac{2*Precision*Sensitivity}{Precision+Sensitivity}.$$ (21)

$$Kappa\;Statistics(KI)=\frac{\left(percent\;agreement\;observed)-(percent\;agreement\;expected\;by\;chance\;alone\right)}{100-(percent\;expected\;by\;chance\;alone)}.$$ (22)

## 3. Results and discussion

Sleep is important for maintaining healthy brain functions and cognition. It is crucial for high level cognition, maintenance, and restoration of physiological functions. In neural processing the slow waves represent association with the memory and removal of waste products through cerebrospinal fluid in the brain [39]. The effectiveness of the sleep apnea dataset was examined in this work, and the proposed categorization of sleep apnea was assessed using metrics such as accuracy, specificity, sensitivity, F1 score, and kappa statistics.

**Fig 5:**
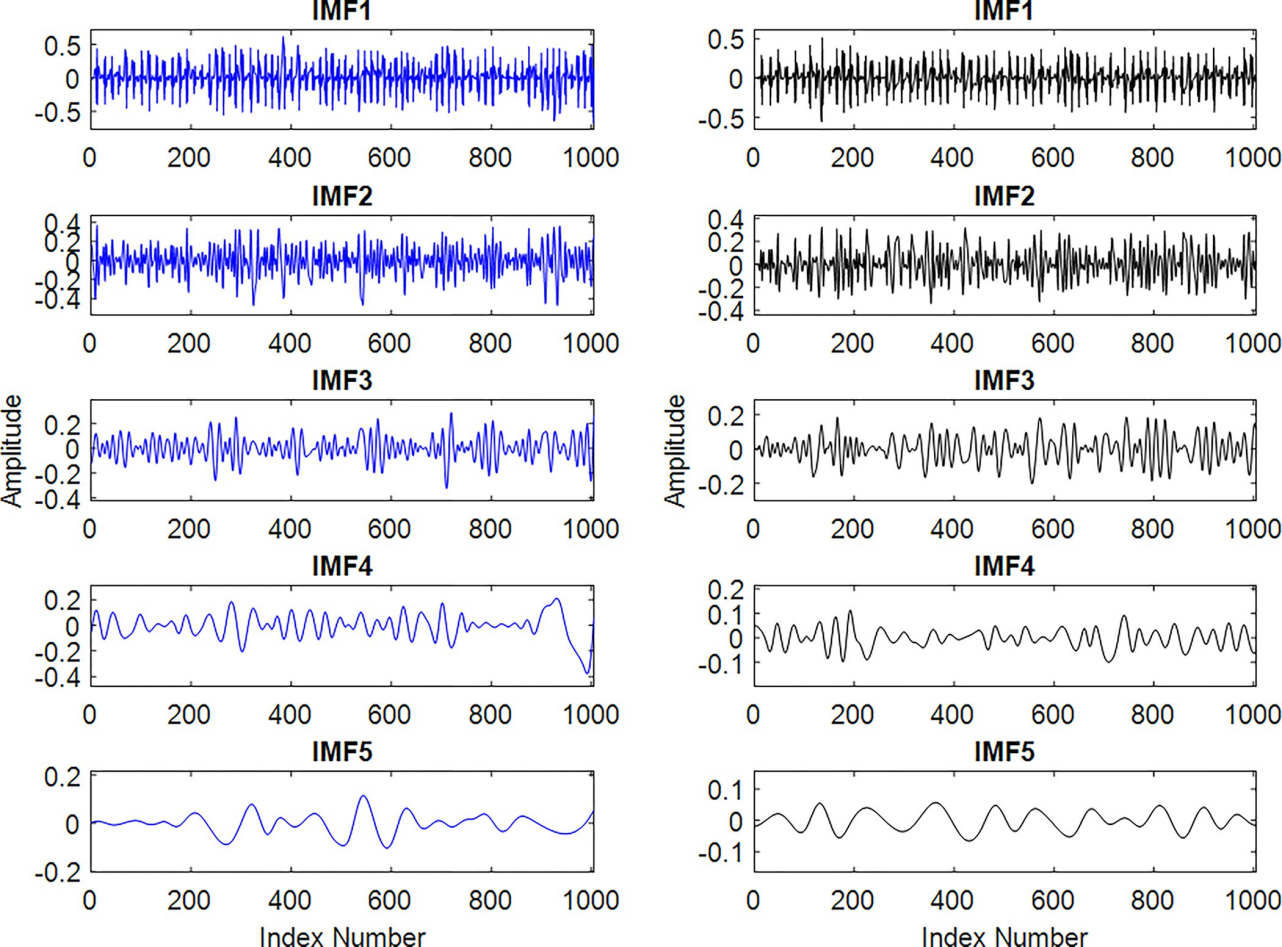
The first five IMFs of *d2s* from Fig 3. For the ECG normal (left-column) and for ECG apnea (right-column).

Firstly, the ECG signals are divided in sub-bands and onward based on the frequency content four sub-bands, *a4*, *d4*, *d3* and *d2*, are selected. In next step from each selected sub-band five IMFs are extracted. Examples of these IMFs, obtained for the *d2* of considered instances of apnea and normal ECG segments are shown in Fig 5.

Afterward, three features are extracted from each IMF using Hjorth parameters. In next step, mined features from each sub-band are concatenated. In this manner each sub-band is presented by 15 features.

Three classes are defined as per AHI. AHI is the total number of apneas and hypopneas per hour of sleep. AHI is used for the identification, quantification of the severity of disease prevalence in normal and clinical individuals.

Table 1 represents the performance of the classification of the devised system for class A versus class B.

**Table 1:** Performance of the apnea classification scheme using various evaluation measures for the class A verses class B problem.

Sub-bands	Class A versus Class B				
	Acc	F1	Sen	Spec	KI
Delta (*a4*) 0–4 Hz	83.45%	83.56%	82.67%	83.78%	76.00%
Theta (*d4*) 4–8 Hz	84.67%	85.67%	84.12%	86.12%	67.00%
Alpha (*d3*) 8–16 Hz	89.78%	81.56%	93.67%	96.67%	89.00%
Beta (*d2*) 16–32 Hz	86.67%	85.67%	84.67%	86.34%	67.00%

Table 1 shows that the highest Acc score of 89.78% is secured for the sub-band Alpha. The second highest Acc of 86.67% is obtained for the sub-band Beta and the lowest Acc of 83.45% is attained for the sub-band Delta. The highest F1-score of 85.67% is secured for the sub-bands Theta and Beta. The second highest F1-score of 83.56% is obtained for the sub-band Delta and the lowest F1-Score of 81.56% is attained for the sub-band Alpha. The highest Sen value of 93.67% is secured for the sub-band Alpha. The second highest Sen of 84.67% is obtained for the sub-band Beta and the lowest Sen value of 82.67% is attained for the sub-band Delta. The highest Spec value of 96.67% is secured for the sub-band Alpha. The second highest Spec of 86.34% is obtained for the sub-band Beta and the lowest Spec of 83.78% is attained for the sub-band Delta. The highest KI of 89.00% is secured for the sub-band Alpha. The second highest KI of 76.00% is obtained for the sub-band Delta and the lowest KI of 67.00% is attained for the sub-bands Theta and Beta.

Table 1, represents moderate performance measure scores for the sub-bands Delta, Theta, and Beta. A significant level of raise is observed for the case of sub-band Alpha. Alpha subsets have a frequency range from 8 Hz to 16 Hz, these waves are emitted in a state of mental and physical relaxation (awake but not involved in neural processing). The SA detection in class A versus class B, shows the measure which are highest in Alpha sub-band are the Acc, Sen, Spec, and KI that estimates wake-sleep transition. Alpha waves occurs more strongly in the occipital region of the brain and can also be recorded from the parietal and frontal region of the scalp. In normal state of deep sleep condition, the alpha waves disappear [40].

Table 2 represents the performance of the classification of the devised system for class A versus class C. It shows that the highest Acc score of 98.12% is secured for the sub-band Delta. The second highest Acc of 89.67% is obtained for the sub-band Theta and the lowest Acc of 71.12% is attained for the sub-band Beta. The highest F1-score of 97.67% is secured for the sub-bands Delta. The second highest F1-score of 85.78% is obtained for the sub-band Theta and the lowest F1-Score of 69.67% is attained for the sub-band Beta. The highest Sen value of 97.89% is secured for the sub-band Delta. The second highest Sen of 84.89% is obtained for the sub-band Theta and the lowest Sen of 63.89% is attained for the sub-band Beta. The highest Spec value of 97.45% is secured for the sub-band Delta. The second highest Spec of 83.89% is obtained for the sub-band Theta and the lowest Spec of 69.56% is attained for the sub-band Beta. The highest KI of 95.00% is secured for the sub-band Delta. The second highest KI of 81.00% is obtained for the sub-band Theta and the lowest KI of 67.00% is attained for the sub-band Beta.

**Table 2:** Performance of the apnea classification scheme using various evaluation measures for the class A versus class C problem.

Sub-bands	Class A versus Class C				
	Acc	F1	Sen	Spec	KI
Delta (*a4*) 0–4 Hz	98.12%	97.67%	97.89%	97.45%	95.00%
Theta (*d4*) 4–8 Hz	89.67%	85.78%	84.89%	83.89%	81.00%
Alpha (*d3*) 8–16 Hz	82.67%	80.34%	80.56%	80.45%	71.00%
Beta (*d2*) 16–32 Hz	71.12%	69.67%	63.89%	69.56%	67.00%

Table 2, represents moderate performance measure scores for the sub-bands Theta, Alpha, and Beta. A significant level of raise is observed for the case of sub-band Delta. Delta subsets have a frequency range from 0 Hz to 4 Hz, these waves are slow and an individual can experience twitching hands, legs and can experience nightmares. The SA detection in class A versus class C, shows the measure which are highest in Delta sub-band are the Acc, Sen, Spec, and KI that estimates twitching hands, legs or nightmares. Delta waves are evident in the waking individual when parts of the brain have been harmed by inflammation, a tumour, or vascular blockage. Delta waves are normally occur during deep sleep [40].

Table 3 represents the performance of the classification of the devised system for class B versus class C.

**Table 3:** Performance of the apnea classification scheme using various evaluation measures for the class B versus class C problem.

Subsets	Class B versus Class C				
	Acc	F1	Sen	Spec	KI
Delta (*a4*) 0–4 Hz	89.78%	89.67%	86.89%	89.45%	87.00%
Theta (*d4*) 4–8 Hz	85.45%	85.67%	84.78%	84.89%	67.00%
Alpha (*d3*) 8–16 Hz	70.67%	71.45%	70.34%	69.56%	67.00%
Beta (*d2*) 16–32 Hz	98.00%	98.00%	97.67%	97.67%	97.00%

Table 3 shows that the highest Acc score of 98.00% is secured for the sub-band Beta. The second highest Acc of 89.78% is obtained for the sub-band Delta and the lowest Acc of 70.67% is attained for the sub-band Alpha. The highest F1-score of 98.00% is secured for the sub-bands Beta. The second highest F1-score of 89.67% is obtained for the sub-band Delta and the lowest F1-Score of 71.45% is attained for the sub-band Alpha. The highest Sen value of 97.67% is secured for the sub-band Beta. The second highest Sen of 86.89% is obtained for the sub-band Delta and the lowest sen of 70.34% is attained for the sub-band Alpha. The highest Spec value of 97.67% is secured for the sub-band Beta. The second highest Spec of 89.45% is obtained for the sub-band Delta and the lowest Spec of 69.56% is attained for the sub-band Alpha. The highest KI of 97.00% is secured for the sub-band Beta. The second highest KI of 87.00% is obtained for the sub-band Delta and the lowest KI of 67.00% is attained for the sub-bands Theta and Alpha.

Table 3, represents moderate performance measure scores for the sub-bands Delta, Theta and Alpha. A significant level of raise is observed for the case of sub-band Beta. Beta subsets have a frequency range from 16 Hz to 32 Hz, these waves replaces alpha waves during attention to task or stimuli. The SA detection in class B versus class C, shows the measure which are highest in Beta sub-band are the Acc, Sen, Spec, and KI [40].

Table 4 represents the performance of the classification of the devised system for Apnea class versus Normal class.

**Table 4:** Performance of the apnea classification scheme using various evaluation measures for the class apnea verses class normal problem.

Subsets	Class Apnea versus Normal				
	Acc	F1	Sen	Spec	KI
Delta (*a4*) 0–4 Hz	81.67%	82.67%	82.67%	92.67%	98.00%
Theta (*d4*) 4–8 Hz	71.23%	72.67%	80.67%	80.34%	82.00%
Alpha (*d3*) 8–16 Hz	78.89%	78.67%	85.34%	85.23%	84.00%
Beta (*d2*) 16–32 Hz	71.67%	73.56%	82.45%	92.56%	96.00%

Table 4 shows that the highest Acc score of 81.67% is secured for the sub-band Delta. The second highest Acc of 78.89% is obtained for the sub-band Alpha and the lowest Acc of 71.23% is attained for the sub-band Theta. The highest F1-score of 82.67% is secured for the sub-bands Delta. The second highest F1-score of 78.67% is obtained for the sub-band Alpha and the lowest F1-Score of 72.67% is attained for the sub-band Theta. The highest Sen value of 85.34% is secured for the sub-band Alpha. The second highest Sen of 82.67% is obtained for the sub-band Delta and the lowest Sen of 80.67% is attained for the sub-band Theta. The highest Spec value of 97.67% is secured for the sub-band Delta. The second highest Spec of 92.56% is obtained for the sub-band Beta and the lowest Spec of 80.34% is attained for the sub-band Theta. The highest KI of 98.00% is secured for the sub-band Delta. The second highest KI of 96.00% is obtained for the sub-band Beta and the lowest KI of 82.00% is attained for the sub-bands Theta.

Table 4, represents moderate performance measure scores for the sub-bands Theta, Alpha and Beta. A significant level of raise is observed for the case of sub-band Delta. Delta subsets have a frequency range from 0 Hz to 4 Hz, these waves are slow and an individual can experience deep sleep in all age of patients. The SA detection in class Apnea versus Normal, shows the measure which are highest in Delta sub-band are the Acc, Sen, Spec, and KI that estimates deep sleep or nightmares. Delta waves are evident in the waking individual when parts of the brain have been harmed by inflammation, a tumour, or vascular blockage [40].

In order to avoid bias, the performance is checked for a variety of assessment metrics, including "Accuracy" (Acc), "F1-score," "Specificity," (Spec),"Sensitivity,"(Sen), and "Kappa index,"(KI).

The results demonstrated that the classifier used in this study shows superior performance with feature space designed by multirate processing, hybrid wavelet-empirical mode decomposition and Hjorth features extraction. Our method has shown better classification accuracies with number of features. Class A Verses Class C attained highest classification accuracy of 98.12% showing wavelet dependent feature can discriminate normal ECG and apnea which provides a better classification metrics than many of the existing technologies. The subset using Beta band of class B Verses Class C has shown the best overall performance in terms of Accuracy,sensitivity,Specificity,F1 and Kappa index where the achieved values were 98, 98,97.67.97.67 and 97 for accuracy,F1,sensitivity, specificity and Kappa index respectively.

Comparative statement with contemporary research work is as shown in Table 5. Which makes it obvious that the proposed strategy performs better or similar to its existing counterparts.

**Table 5:** Comparison with state of the art counter parts.

Study	Signals	Feature Extraction	Classifier	Remark	Accuracy
Fatimah et al., 2020 [41]	ECG	Fourier decomposition (FD) + sub bands statistical features	SVM	Binary classification	92.59%
Mencar et al., 2020 [11]	Demographic characteristics, Spirometry values and symptoms	PCA+ Communality Index	SVM	Limited size dataset	65%
Kristiansen et al., 2021 [12]	C,A,N,O	NA	Recurrent NN Bidirectional LSTM with attention (BIWALSTM)	Low Cost Sensor can be used	89.41%
Kandala et al., 2021 [13]	ECG	DWT +Correlation based Feature Selection with PSO	RF with 10 fold cross validation	Sensitivity of Proposed approach is slightly less. Data has to be validated for the subjects with sleep disorders.	90%
Sheta et al., 2021 [14]	ECG	Temporal and Spectral feature Extraction	CNNLSTM		86.25%
Xie et al., 2012 [15]	SPO_2_		Bagging with REPTree		84.40%
Bahrami et al., 2022 [16]	ECG	PCA	ZFNet-BiLSTM	Lesser number of mild and moderate SA patients and the lack of CSA episodes are the main limitations of the database	88.13%
Wang et al., 2019 [17]	ECG	Time Domain +Frequency Domain features	TW-MLP		87.3%
Tuncer et al., 2019 [18]	PTT	Alexnet +VGG-16	SVM	Complex internal mechanism	92.78%
Urtnasan et al., 2018 [19]	ECG	1D convolution	CNN	Multiclass classification	90.8%
**Proposed approach**	**ECG**	**Hybrid Wavelet-EMD decomposition + Hjorth features extraction from IMFs**	**MLPNN**	**Binary classification (class A Verses class B)**	**89.78%**
				**Binary classification (class A Verses class C)**	**98.12%**
				**Binary classification (class B Verses class C)**	**98.00%**
				**Binary classification (class Apnea Verses class Normal)**	**81.67%**

## 4. Conclusion

This paper presents a simple and accurate automated computer-aided approach for the sleep apnea detection. The pertinent Hjorth features are mined from the wavelet driven intrinsic mode functions of the intended ECG signals. Onward, these features are employed in categorizing the intended ECG segments for apnea identification. The devised method secured a classification accuracy of 98%, showing that this approach can effectively discriminate normal and apnea ECG signals and can provide an accurate or comparable classification metrics compared to the existing counterparts. In future, the performance of proposed method will to evaluated on other potential datasets prior to its use in clinical practice. Other multiclass measures are also suggested to be evaluated as future direction of this work. Data variability can be addressed through data augmentation. Exploring advanced signal processing techniques, especially those tailored for the ECG signals, might yield better features for distinguishing the apnea events. Combining ECG data with other physiological signals, such as respiratory or electroencephalogram, could provide a more robust categorization and comprehensive view of sleep apnea events.

## References

[1] Benjafield A. V. et al., “Estimation of the global prevalence and burden of obstructive sleep apnoea: a literature-based analysis,” *Lancet Respir Med*, vol. 7, no. 8, pp. 687–698, Aug. 2019, doi: 10.1016/S2213-2600(19)30198-5 31300334 PMC7007763

[2] Kabir A., Ifteqar S., and Bhat A., “Obstructive sleep apnea in adults,” *Hospital Practice*, vol. 41, no. 4, pp. 57–65, 2013. doi: 10.3810/hp.2013.10.1081 24145590

[3] Berry R. B., Brooks R., Gamaldo C. E., Harding S. M., Marcus C., and Vaughn B. V., “The AASM manual for the scoring of sleep and associated events,” *Rules*, *Terminology and Technical Specifications*, *Darien*, *Illinois*, *American Academy of Sleep Medicine*, vol. 176, p. 2012, 2012.

[4] Dempsey J. A., Veasey S. C., Morgan B. J., and O’Donnell C. P., “Pathophysiology of Sleep Apnea,” *Physiological Reviews*, Jan. 2010, doi: 10.1152/physrev.00043.2008 20086074 PMC3970937

[5] Jordan A. S. et al., “The influence of gender and upper airway resistance on the ventilatory response to arousal in obstructive sleep apnoea in humans,” *The Journal of physiology*, vol. 558, no. 3, pp. 993–1004, 2004. doi: 10.1113/jphysiol.2004.064238 15218069 PMC1665031

[6] Eikermann M. et al., “The influence of aging on pharyngeal collapsibility during sleep,” *Chest*, vol. 131, no. 6, pp. 1702–1709, 2007. doi: 10.1378/chest.06-2653 17413053 PMC2278166

[7] Kirkness J. P., Madronio M., Stavrinou R., Wheatley J. R., and Amis T. C., “Surface tension of upper airway mucosal lining liquid in obstructive sleep apnea/hypopnea syndrome,” *Sleep*, vol. 28, no. 4, pp. 457–463, 2005. doi: 10.1093/sleep/28.4.457 16171290

[8] Barkdull G. C., Kohl C. A., Patel M., and Davidson T. M., “Computed Tomography Imaging of Patients With Obstructive Sleep Apnea,” *The Laryngoscope*, vol. 118, no. 8, pp. 1486–1492, 2008, doi: 10.1097/MLG.0b013e3181782706 18528305

[9] Eckert D. J. and Malhotra A., “Pathophysiology of adult obstructive sleep apnea,” *Proceedings of the American thoracic society*, vol. 5, no. 2, pp. 144–153, 2008. doi: 10.1513/pats.200707-114MG 18250206 PMC2628457

[10] Mezzanotte W. S., Tangel D. J., and White D. P., “Waking genioglossal electromyogram in sleep apnea patients versus normal controls (a neuromuscular compensatory mechanism).,” *The Journal of clinical investigation*, vol. 89, no. 5, pp. 1571–1579, 1992. doi: 10.1172/JCI115751 1569196 PMC443031

[11] Mencar C. et al., “Application of machine learning to predict obstructive sleep apnea syndrome severity,” *Health informatics journal*, vol. 26, no. 1, pp. 298–317, 2020. doi: 10.1177/1460458218824725 30696334

[12] Kristiansen S. et al., “Machine Learning for Sleep Apnea Detection with Unattended Sleep Monitoring at Home,” *ACM Trans*. *Comput*. *Healthcare*, vol. 2, no. 2, p. 14:1–14:25, Feb. 2021, doi: 10.1145/3433987

[13] Kandala N. V. P. S. Rajesh R. Dhuli, and Kumar T. S., “Obstructive sleep apnea detection using discrete wavelet transform-based statistical features,” *Computers in Biology and Medicine*, vol. 130, p. 104199, Mar. 2021, doi: 10.1016/j.compbiomed.2020.104199 33422885

[14] Sheta A. et al., “Diagnosis of Obstructive Sleep Apnea from ECG Signals Using Machine Learning and Deep Learning Classifiers,” *Applied Sciences*, vol. 11, no. 14, Art. no. 14, Jan. 2021, doi: 10.3390/app11146622

[15] Xie B. and Minn H., “Real-Time Sleep Apnea Detection by Classifier Combination,” *IEEE Transactions on Information Technology in Biomedicine*, vol. 16, no. 3, pp. 469–477, May 2012, doi: 10.1109/TITB.2012.2188299 22353404

[16] Bahrami M. and Forouzanfar M., “Sleep Apnea Detection From Single-Lead ECG: A Comprehensive Analysis of Machine Learning and Deep Learning Algorithms,” *IEEE Transactions on Instrumentation and Measurement*, vol. 71, pp. 1–11, 2022, doi: 10.1109/TIM.2022.3151947

[17] Wang T., Lu C., and Shen G., “Detection of Sleep Apnea from Single-Lead ECG Signal Using a Time Window Artificial Neural Network,” *Biomed Res Int*, vol. 2019, p. 9768072, Dec. 2019, doi: 10.1155/2019/9768072 31950061 PMC6948296

[18] Arslan Tuncer S., Akılotu B., and Toraman S., “A deep learning-based decision support system for diagnosis of OSAS using PTT signals,” *Medical Hypotheses*, vol. 127, pp. 15–22, Jun. 2019, doi: 10.1016/j.mehy.2019.03.026 31088639

[19] Urtnasan E., Park J.-U., and Lee K.-J., “Multiclass classification of obstructive sleep apnea/hypopnea based on a convolutional neural network from a single-lead electrocardiogram,” *Physiological measurement*, vol. 39, no. 6, p. 065003, 2018. doi: 10.1088/1361-6579/aac7b7 29794342

[20] Xue Y., Cao J., Tian R., Du H., and Shu Y., “Application of the empirical mode decomposition and wavelet transform to seismic reflection frequency attenuation analysis,” *Journal of Petroleum Science and Engineering*, vol. 122, pp. 360–370, 2014.

[21] Penzel T., McNames J., Murray A., de Chazal P., Moody G., and Raymond B., “Systematic comparison of different algorithms for apnoea detection based on electrocardiogram recordings,” *Med Biol Eng Comput*, vol. 40, no. 4, pp. 402–407, Jul. 2002, doi: 10.1007/BF02345072 12227626

[22] Penzel T., Moody G. B., Mark R. G., Goldberger A. L., and Peter J. H., “The apnea-ECG database,” in *Computers in Cardiology 2000*. *Vol*.*27 (Cat*. *00CH37163)*, Cambridge, MA, USA: IEEE, 2000, pp. 255–258. doi: 10.1109/CIC.2000.898505

[23] “Detecting and Quantifying Apnea Based on the ECG: The PhysioNet/Computing in Cardiology Challenge 2000 v1.0.0.” https://physionet.org/content/challenge-2000/1.0.0/ (accessed Sep. 06, 2023).

[24] Malghan P. G. and Hota M. K., “Grasshopper optimization algorithm based improved variational mode decomposition technique for muscle artifact removal in ECG using dynamic time warping,” *Biomedical Signal Processing and Control*, vol. 73, p. 103437, 2022.

[25] Qaisar S. M., Khan S. I., Srinivasan K., and Krichen M., “Arrhythmia classification using multirate processing metaheuristic optimization and variational mode decomposition,” *Journal of King Saud University-Computer and Information Sciences*, 2022.

[26] Saritha Raj K., Rajesh Kumar P., and Satyanarayana M., “Capricious Digital Filter Design and Implementation Using Baugh–Wooley Multiplier and Error Reduced Carry Prediction Approximate Adder for ECG Noise Removal Application,” *Circuits*, *Systems*, *and Signal Processing*, pp. 1–23, 2023.

[27] Mian Qaisar S. and Hussain S. F., “An effective arrhythmia classification via ECG signal subsampling and mutual information based subbands statistical features selection,” *Journal of Ambient Intelligence and Humanized Computing*, pp. 1–15, 2021.

[28] Qaisar S. M., Khan S. I., Dallet D., Tadeusiewicz R., and Pławiak P., “Signal-piloted processing metaheuristic optimization and wavelet decomposition based elucidation of arrhythmia for mobile healthcare,” *Biocybernetics and Biomedical Engineering*, vol. 42, no. 2, pp. 681–694, 2022.

[29] Pachori R. B., “Discrimination between Ictal and Seizure-Free EEG Signals Using Empirical Mode Decomposition,” *Research Letters in Signal Processing*, 2008, doi: 10.1155/2008/293056

[30] Pachori R. B. and Bajaj V., “Analysis of normal and epileptic seizure EEG signals using empirical mode decomposition,” *Computer Methods and Programs in Biomedicine*, 2011, doi: 10.1016/j.cmpb.2011.03.009 21529981

[31] Salankar N. et al., “Impact of Music in Males and Females for Relief from Neurodegenerative Disorder Stress,” *Contrast Media & Molecular Imaging*, vol. 2022, p. e3080437, Apr. 2022, doi: 10.1155/2022/3080437 35494208 PMC9019444

[32] Hjorth B., “EEG analysis based on time domain properties,” *Electroencephalography and Clinical Neurophysiology*, vol. 29, no. 3, pp. 306–310, Sep. 1970, doi: 10.1016/0013-4694(70)90143-4 4195653

[33] Vidaurre C., Krämer N., Blankertz B., and Schlögl A., “Time Domain Parameters as a feature for EEG-based Brain–Computer Interfaces,” *Neural Networks*, vol. 22, no. 9, pp. 1313–1319, 2009, doi: 10.1016/j.neunet.2009.07.020 19660908

[34] Safi M. S. and Safi S. M. M., “Early detection of Alzheimer’s disease from EEG signals using Hjorth parameters,” *Biomedical Signal Processing and Control*, vol. 65, p. 102338, Mar. 2021, doi: 10.1016/j.bspc.2020.102338

[35] Kingma D. P. and Ba J., “Adam: A method for stochastic optimization,” *arXiv preprint arXiv*:*1412*.6980, 2014.

[36] Salankar N., Koundal D., Chakraborty C., and Garg L., “Automated attention deficit classification system from multimodal physiological signals,” *Multimedia Tools and Applications*, Feb. 2022, doi: 10.1007/s11042-022-12170-1

[37] Darmawahyuni A., Nurmaini S., Yuwandini M., Muhammad Naufal Rachmatullah F. Firdaus, and Tutuko B., “Congestive heart failure waveform classification based on short time-step analysis with recurrent network,” *Informatics in Medicine Unlocked*, vol. 21, p. 100441, Jan. 2020, doi: 10.1016/j.imu.2020.100441

[38] Subasi A., *Practical guide for biomedical signals analysis using machine learning techniques*: *A MATLAB based approach*. Academic Press, 2019.

[39] Fultz N. E. et al., “Coupled electrophysiological, hemodynamic, and cerebrospinal fluid oscillations in human sleep,” *Science*, vol. 366, no. 6465, pp. 628–631, 2019. doi: 10.1126/science.aax5440 31672896 PMC7309589

[40] Moini J. and Piran P., “Chapter 6—Cerebral cortex,” in *Functional and Clinical Neuroanatomy*, Moini Jand Piran P., Eds., Academic Press, 2020, pp. 177–240. 10.1016/B978-0-12-817424-1.00006-9.

[41] Fatimah B., Singh P., Singhal A., and Pachori R. B., “Detection of apnea events from ECG segments using Fourier decomposition method,” *Biomedical Signal Processing and Control*, vol. 61, p. 102005, Aug. 2020, doi: 10.1016/j.bspc.2020.102005

